# Monitoring Extracellular Vesicle Cargo Active Uptake by Imaging Flow Cytometry

**DOI:** 10.3389/fimmu.2018.01011

**Published:** 2018-05-24

**Authors:** Yifat Ofir-Birin, Paula Abou karam, Ariel Rudik, Tal Giladi, Ziv Porat, Neta Regev-Rudzki

**Affiliations:** ^1^Department of Biomolecular Sciences, Faculty of Biochemistry, Weizmann Institute of Science, Rehovot, Israel; ^2^Flow Cytometry Unit, Life Sciences Core Facilities, Weizmann Institute of Science, Rehovot, Israel

**Keywords:** extracellular vesicles, imaging flow cytometry, malaria, *Plasmodium falciparum*, vesicle uptake

## Abstract

Extracellular vesicles are essential for long distance cell–cell communication. They function as carriers of different compounds, including proteins, lipids and nucleic acids. Pathogens, like malaria parasites (*Plasmodium falciparum, Pf*), excel in employing vesicle release to mediate cell communication in diverse processes, particularly in manipulating the host response. Establishing research tools to study the interface between pathogen-derived vesicles and their host recipient cells will greatly benefit the scientific community. Here, we present an imaging flow cytometry (IFC) method for monitoring the uptake of malaria-derived vesicles by host immune cells. By staining different cargo components, we were able to directly track the cargo’s internalization over time and measure the kinetics of its delivery. Impressively, we demonstrate that this method can be used to specifically monitor the translocation of a specific protein within the cellular milieu upon internalization of parasitic cargo; namely, we were able to visually observe how uptaken parasitic *Pf*-DNA cargo leads to translocation of transcription factor IRF3 from the cytosol to the nucleus within the recipient immune cell. Our findings demonstrate that our method can be used to study cellular dynamics upon vesicle uptake in different host–pathogen and pathogen–pathogen systems.

## Introduction

Extracellular vesicles (EVs) are membrane-surrounded structures that are secreted by cells into the intercellular environment. EVs shuttle lipids, proteins, RNA, DNA, and other metabolites between cells and tissues. They diverge into two main subgroups according to their cellular origin: microvesicles (200–1,000 nm in diameter) are shed from the plasma membrane, whereas exosomes, which are smaller in size (40–200 nm in diameter) originate from the endosome as intraluminal vesicles enclosed within multivesicular bodies (1–3). Over the past decade, it has become clear that most, if not all, organisms utilize this evolutionary conserved mechanism for cell-to-cell communication within and between populations [reviewed in Ref. (4–6)]. A key element in this communication process is EV uptake by recipient cells, which generally includes endocytosis, phagocytosis, and micropinocytosis [reviewed in Ref. (7)]. Although several pathways have been suggested, the specific molecular events that regulate EV translocation and uptake by target cells remain almost entirely unknown. Therefore, there is a need to develop new techniques to study these events.

Pathogens, in particular, have found EVs to be a useful tool for evading and manipulating the immune response, ultimately succeeding in infecting new susceptible hosts (4, 8–10). Parasites, for instance, are known for their remarkable ability to avoid the host immune system, yet in many cases the mechanisms that underlie these processes are still unknown. *Plasmodium falciparum* (*Pf*), one of the most deadly species of *Plasmodium*, causes malaria in humans. Recent studies have revealed that the intracellular malaria parasites secrete EVs from the host cell to deliver multiple components that promote cell communication (11–16). Importunately, it was shown that parasitic EV-DNA is transferred into the host cytosol, where it is detected by the STING-dependent cytosolic DNA sensing pathway to modulate host gene induction from a distance. Upon sensing *Pf*-EV-DNA in the cytoplasm, the protein STING becomes active and prompts a chain of events that includes the phosphorylation of kinase TBK1 and transcription factor IRF3. Phosphorylated IRF3 (pIRF3) then enters the nucleus to induce the transcription of genes, including type I IFN genes (16).

Since EVs harbor promising clinical applications (2, 17) both as diagnostic tools and as a drug delivery mechanism (18), high-throughput technologies for detecting EVs in a population-based manner are warranted (19, 20). Such demands for advanced and robust tools have led to adaptations of large-scale imaging approaches, including imaging flow cytometry (IFC) (21–23).

Imaging flow cytometry combines the speed and high-throughput of conventional flow cytometry with the information-rich imagery of microscopy. These distinct abilities enable IFC to rapidly acquire high-quality multispectral images (24–26). This technique allows the measurement not only of fluorescence levels, but also of the pixel distribution and cellular localization, such as distinguishing between homogenous and speckled staining and the co-localization of different markers, respectively. When using conventional flow cytometry, the detection of individual EVs is often misleading due to their nano-size, which falls within the range of electronic noise. IFC overcomes this drawback, since the ability to measure single pixel intensities enables it to even detect fluorescent particles that are smaller than the diffraction limit (23, 24, 27–29).

Here, we demonstrate that IFC can be used as an accurate large-scale method for tracking the dynamics of the uptake of individual types of cargo components (RNA, proteins, and lipids). We further utilized the system of activated IRF3 translocation as a platform for demonstrating the capability of IFC to specifically monitor protein translocation within target cells. Using IFC, we were able to determine the kinetics of the translocation of pIRF3 from the cytosol into the nucleus following insertion of *Pf*-DNA cargo (24 h analysis).

This powerful approach paves the way not only to measuring the process of vesicle internalization by different recipient cells, but also to directly studying activated protein movement and, thus, further investigating related cellular signaling events.

## Method

### Parasite Line and Culture

The NF54 parasite line was obtained from the Malaria Research Reference Reagent Resource Center (MR4). Parasites were maintained in culture in O+ or A+ erythrocytes at 4% hematocrit in RPMI-HEPES supplemented with 0.5% (w/v) AlbumaxII (Invitrogen) as previously described (30).

### EV Isolation and Fluorescence Staining

Extracellular vesicles were isolated from the NF54 strain in a high parasitemia (approximately 8%) of *Pf*-infected red blood cells (RBCs) culture using a Beckman OPTIMA90X ultracentrifuge with a TI70 rotor, as previously described (31). The pellet was resuspended in PBS^−/−^, and the purified EVs were stained according to the manufacturer’s protocol with slight modifications, as described below. We used several fluorescent stains for the different vesicle compounds: thiazole orange (TO) (Sigma Aldrich) for RNA-cargo, Ghost Dye UV (GO) (Tonbo bioscience) for protein cargo, and DiI, DiD, or DiO (Thermo Fisher Scientific) for lipid cargo. For the double-staining assay, EVs were stained using a combination of DiI and GO; DiD and GO; DiD and TO; or TO and GO. The stains were incubated with EVs at a 1 µl/ml ratio at 37°C for 30 min. Labeled vesicles were then washed in ice-cold PBS and precipitated again in an ultracentrifuge at 37k RPM over night. Next, the vesicle pellet was washed and resuspended in PBS^−/−^, and the size and concentration of the labeled vesicles were measured by NanoSight ns300 with the associated laser (32).

### EV Uptake Into Monocytes

Monocyte cells of the THP-1 cell line were cultured (33) overnight in RPMI1640+ l-glutamine (Biological Industries Ltd., Beit Ha’Emek, Israel) and 10% FBS (Biological Industries Ltd., Beit Ha’Emek, Israel). Prior to the vesicle treatment, cells were washed in PBS^−/−^, resuspended in RPMI1640+ (Biological Industries Ltd., Beit Ha’Emek, Israel) and plated in 6-well plate, ~1.5*10^6^ cells per well. For EV comparative uptake measurements, THP-1 cells were incubated with an increased relative volume amount of labeled *P. falciparum* infected RBC- derived EVs (0, 10, 50, and 100%) for 5 min before being fixated in 4% PFA for 30 min on ice, washed in PBS and analyzed by IFC (see below).

### IRF3 Translocation Analysis

THP-1 cells were transfected with *P. falciparum* genomic DNA for 5 or 24 h, as was previously done (16). Following transfection, cells were fixed and permeabilized with 4% PFA and 2% sucrose at 4°C for 30 min. Fixated cells were washed and blocked with filtered 5% BSA in PBS for 1 h. Primary antibodies, human IRF3 (Cell signaling #11904 1:200 dilution in 5% BSA PBS) and human pIRF3 (Cell signaling #29047 1:50 dilution in 5% BSA PBS) were incubated overnight and washed three times, for 10 min each time, with 5% BSA PBS. Secondary antibody AlexaFluor^®^488 anti-rabbit antibody (Life technology, 1:200 dilution in 5% BSA PBS) and Hoechst (H6024 SIGMA) were incubated for 30 min and then washed three times, for 10 min each time, with 5% BSA PBS and resuspended in PBS (−/−) before being imaged by IFC (see below).

### Multispectral IFC Analysis

Cells or individual EVs were imaged using a multispectral IFC (ImageStreamX mark II, Amnis Corp., Seattle, WA, USA, Part of MERCK-EMD Millipore). To obtain kinetic measurements, THP-1 cells were kept on ice and EVs stained with TO were added. Samples were immediately introduced into the instrument and the acquisition started approximately 90–150 s afterward. In the direct EV uptake measurements, EVs were labeled and ~1.5*10^8^ EVs were imaged using IFC. The ImageStreamX uses calibration beads that are 3 μm. To exclude these beads from the acquisition, objects were gated according to their area and intensity of the side scatter channel (Ch06) and the uniform bead population was easily identified and eliminated. At least 5 × 10^4^ cells were collected from each sample and data were analyzed using the manufacturer’s image analysis software (IDEAS 6.2; Amnis Corp.). Images were compensated for fluorescent dye overlap by using single-stain controls. THP1 cells were gated for single cells, using the area and aspect-ratio features, and for focused cells using the Gradient RMS feature, as previously described (22). Cropped cells were further eliminated by plotting the cell area of the bright field image against the Centroid X feature (the number of pixels in the horizontal axis from the left corner of the image to the center of the cell mask). EV internalization was evaluated using several features, including the intensity (the sum of the background − subtracted pixel values within the masked area of the image) and max pixel (the largest value of the background − subtracted pixel). For IRF3 nuclear translocation, cells were also gated for DNA positive cells according to the area and intensity of the DNA staining, and cell doublets were further eliminated by plotting the area Vs. the aspect ratio of the nuclear staining. The co-localization of IRF3 with the nuclear image (Hoechst) was calculated using the Similarity feature (log transformed Pearson’s Correlation Coefficient between the two images). Values above 1.5 indicate co-localization.

### Monitoring THP-1 Cell Survival Following Uptake of *Pf*-Derived EVs

THP-1 cells were cultivated as described in the EV uptake subsection. ~1*10^6^ THP-1 cells were incubated with 50*10^6^ EVs for 5 min. The cells were then washed and seeded in 6-well plate and monitored for 72 h, live and dead cells were counted and the media changed every 24 h. The viability was tested using trypan blue (Sigma Aldrich).

## Results

### Monitoring *Pf*-Derived EV-Stained Cargo by IFC

**Figure 1:**
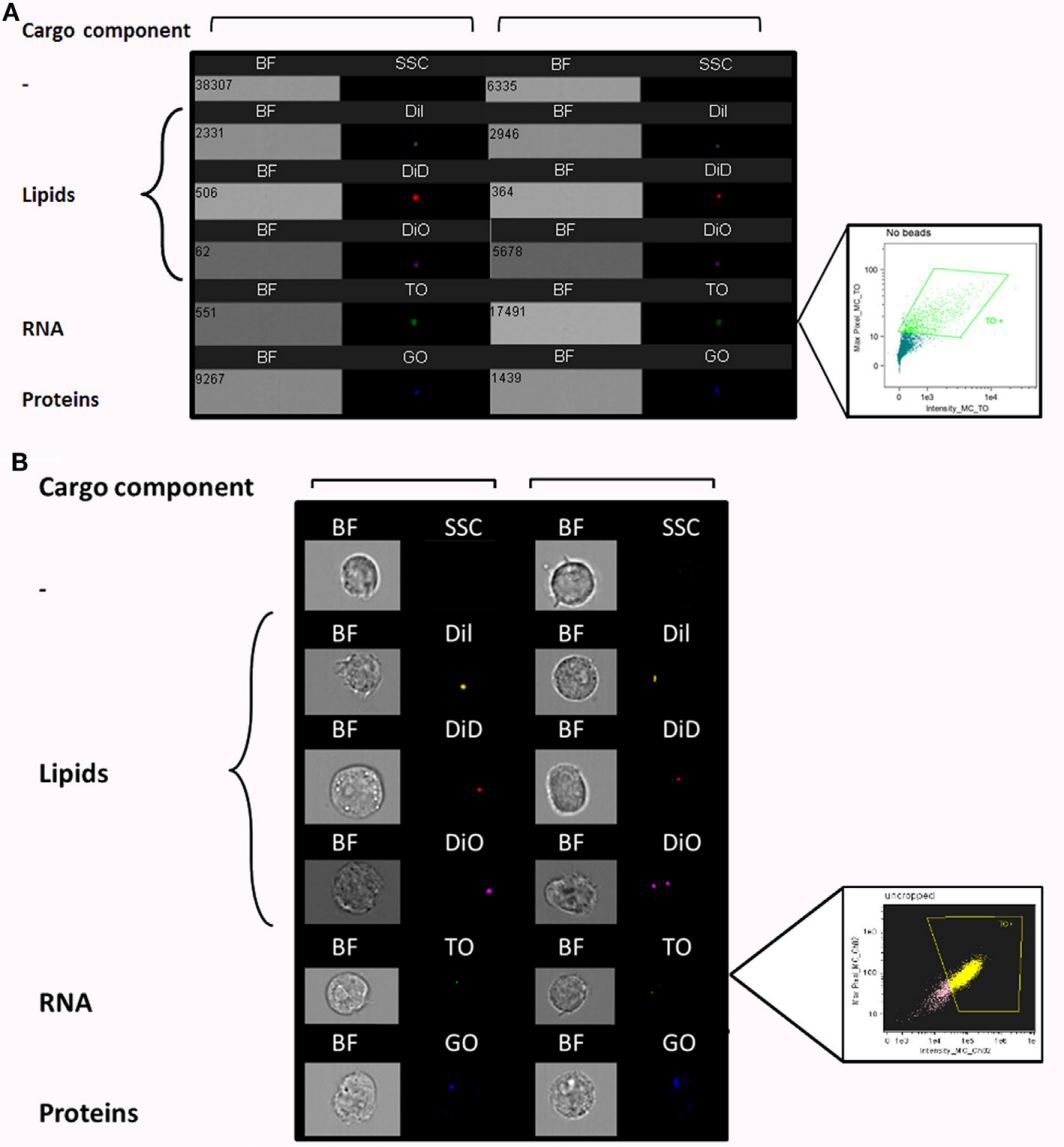
Visualization of *Plasmodium falciparum* (*Pf*)-derived extracellular vesicles (EVs) by imaging flow cytometry (IFC). **(A)** Visualization of single-stained *Pf*-derived EVs by IFC. *Pf*-derived EVs stained with lipid (DiI, DiD, and DiO), RNA [thiazole orange (TO)], or protein (GO) dyes. Insert shows percentage of TO-positive EVs (43%), gated according to unlabeled EVs. Representative results from at least three experiments are shown. Abbreviations: BF, bright field; SSC, side scatter. **(B)** Internalization (uptake) of *Pf-*derived EVs into monocytes as visualized by IFC. EVs were stained with lipid (DiI, DiD, and DiO), RNA (TO), or protein (GO) dyes and then 7.5*10^7^ dyed EVs were introduced into 1.5*10^6^ THP-1 cells for 5 min. The cells were fixated as described in Section “Method” and vesicle uptake was imaged using IFC. EVs are detected as spots inside recipient cells. Insert shows percentage of EV-positive cells (72.5%), gated according to unlabeled EVs. Representative results from at least three experiments are shown. Abbreviations: BF, bright field; SSC, side scatters.

To better characterize the interactions of *Pf*-derived vesicles with host immune target cells, we established an EV uptake assay and were able to fluorescently track labeled vesicles. Since EVs contain proteins, RNA, and lipids, we used different fluorescent stains to specifically label each cargo component in the EVs derived from *Pf*-infected RBCs. TO was used for vesicle RNA, DiI, DiD, and DiO stains for lipids, and Ghost dye for vesicle membrane proteins (Figure 1A). Vesicles imaged with IFC exhibited a clear signal of individual vesicles for each of these cargo-component stains (Figure 1A). The right insert in Figure 1A shows an example of the percentage of RNA (TO)-positive EVs, gated according to unlabeled samples. A Nanosight nc300 particle detector was used to confirm the purity of vesicle production and the fluorescence intensity of the EV population (Figure S1 in Supplementary Material).

Next, we performed a subsequent uptake assay into monocytes (THP-1 cells). Using the different fluorescent stains, we were able to monitor the uptake of RNA, protein, and lipid components within the host immune cells (monocytes) by IFC (Figure 1B). The right insert in Figure 1B shows the percentage of monocytes positive for TO-labeled EVs, gated according to unlabeled samples. While we were able to track the uptake signal of transferred RNA and proteins during the first 40 min of the analysis, the lipid cargo signal was detectable within monocytes only for a very short time period at the start of the incubation period (<5 min). The rapid reduction in the lipid signal may imply that membrane fusion is involved in the uptake mechanism. The internalization of the EVs, however, could be detected only under physiological condition at 37°C and not at 4°C (Figure S3 in Supplementary Material), similar to what was previously shown (16).

### Detecting EV Double-Stained Components Using IFC: An Indication of the Internalization of the Entire Vesicle Into the Host Cells

Detecting and quantifying EVs by IFC have been previously described (12, 23, 24, 27–29, 34, 35). However, due to their small size, detection of individual EVs using bright field only is very limited, as the pixel size is 0.3 µm using the 60× lens. Detection by light scattering using conventional flow cytometry is also limited. Although we can detect sub-micron polystyrene beads, lipid-based vesicles have a lower refractive index than beads (less than 1.4, compared to 1.6 for beads). This results in lower light scattering, placing the signal within the range of background noise. Therefore, to facilitate their detection, fluorescence labeling is needed.

**Figure 2:**
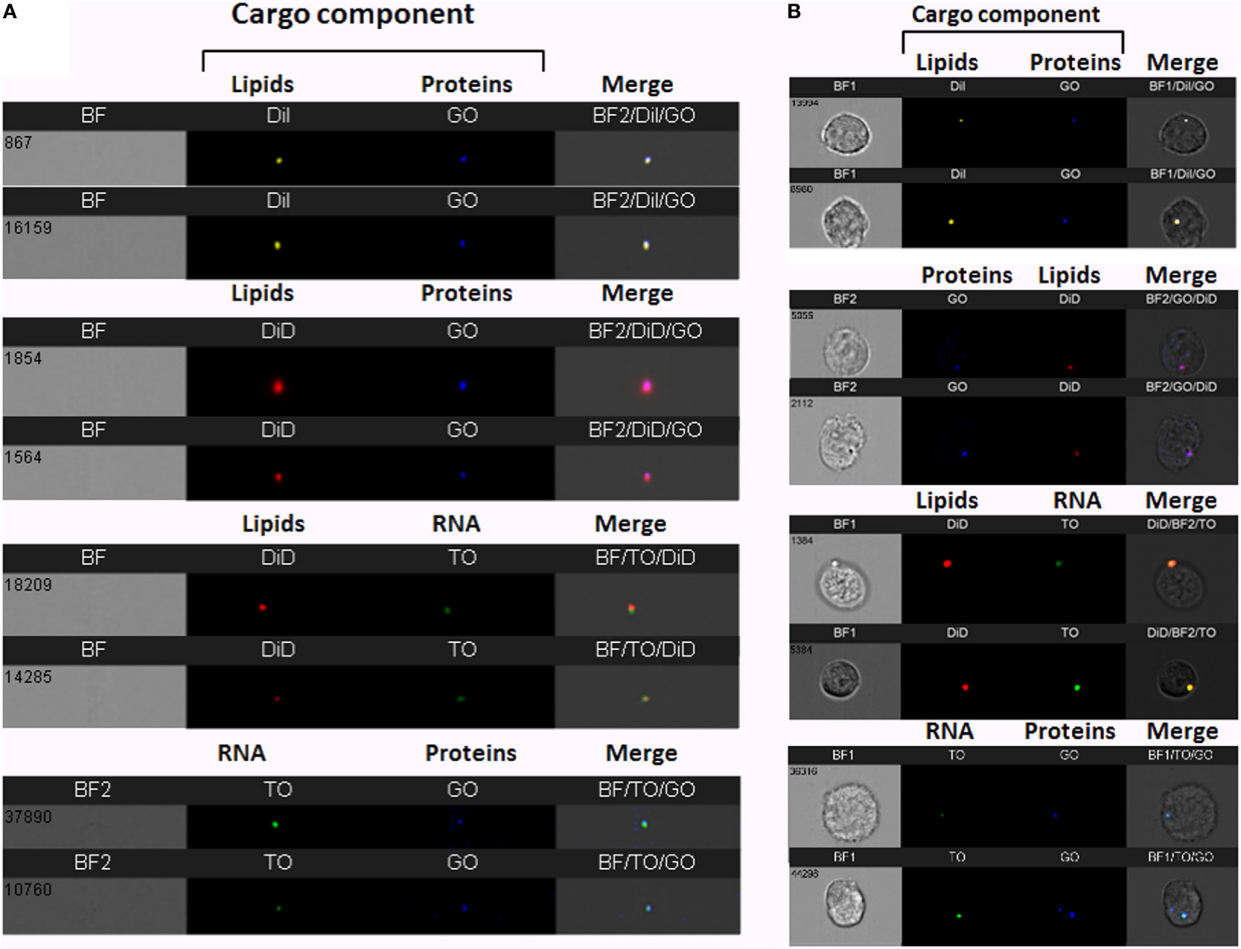
Visualization of double-stained *Plasmodium falciparum* (*Pf*)-derived extracellular vesicles (EVs) by imaging flow cytometry (IFC). **(A)** IFC analysis of double-stained *Pf*-derived EVs. *Pf*-derived EVs co-labeled with different combinations of two stains: for lipids (DiI, DiD, or DiO), RNA [thiazole orange (TO)], and proteins (GO). Representative results from at least three experiments are shown. Abbreviations: BF, bright field; SSC, side scatter **(B)** Uptake assay of *Pf*-derived EVs into monocytes (THP-1). IFC imaging of *Pf*-derived EVs labeled with two stains for lipids (DiI, DiD, or DiO) and/or RNA (TO) and/or proteins (GO) uptaken into THP-1 cells as described in the Section “Method.” Representative results from at least three experiments are shown. Abbreviations: BF, bright field; SSC, side scatter.

To increase cargo detection confidence, we generated double-stained vesicles by co-labeling different components (RNA, proteins, and lipids). Purified *Pf*-infected RBC-derived EVs were co-stained using four different combinations; for instance, co-staining RNA and lipids (Figure 2A). Individual vesicles imaged using IFC were positive for the double-staining (Figure 2A), validating the detection of vesicles containing different molecular components.

We further measured the uptake of the co-stained vesicles within recipient monocytes following 5 min of incubation (Figure 2B). The window of detection within the first 5 min of uptake sufficed to detect the double-staining signals of the different components and these were co-localized in the cell area. The fact that we could detect the different cargo components at the same area (co-localized) within less than 5 min of uptake implies that the entire vesicle is, in fact, inserted into the host cell rather than being fused to the cell’s surface.

**Figure 3:**
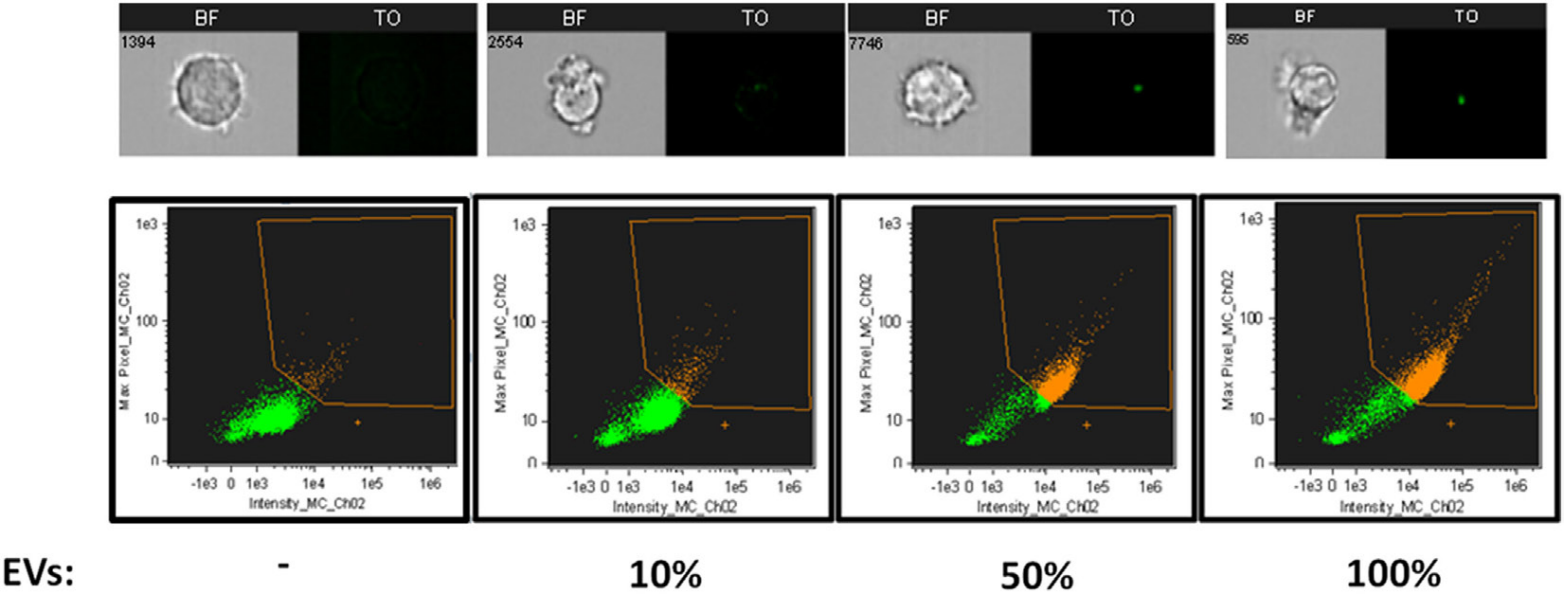
Relative *Plasmodium falciparum* (*Pf*)*-*derived extracellular vesicles (EV) uptake into monocytes. Relative amounts of EVs (100, 50, 10, and 0%) were stained using the same amount of [thiazole orange (TO)] dye. THP-1 cells were incubated with RNA (TO)-labeled EVs for 5 min before being fixated and imaged by imaging flow cytometry. Graphs show the percentage of TO-labeled EV-positive cells, gated according to unlabeled EVs. Representative results from experiments out of three are shown. Abbreviation: BF, bright field.

To verify that indeed the increase in fluorescence intensity is due to EV uptake and not due to auto-fluorescence or dyes aggregates, we incubated THP-1 recipient cells with increasing concentrations of stained EVs and quantified their uptake. As expected, the signal received from the recipient cells increased in line with the amount of vesicles present (Figure 3). This was not a result of dye aggregates, as this increase was not seen when dyes were added to PBS alone, vesicle-free (data not shown). The percentage of THP1 cells positive for TO-labeled EVs was gated according to THP1 cells incubated with unlabeled EVs (Figure 1B).

Imaging flow cytometry can detect fluorescently labeled EVs even at sub-resolution range, since bright enough fluorescence can fill more than one pixel and enable sample detection. Additional removal of artifacts can be done by verifying that the two fluorescent channels co-localize to the same object (Figure 2A), which is not possible in conventional flow cytometry. This demonstrates that IFC can be a useful technique for studying the dynamics of cargo distribution within the cell upon uptake. Specifically, since the EV population originating from the same cells is often heterogeneous, this can lead to diversified uptake mechanisms of target cells and, as a result, can affect cargo destination. Therefore, IFC can be adapted not just to detect cargo internalization, but also to explore the nature of the EV uptake and the internal localization of components.

### Monitoring the Kinetics of the Uptake Into the Host of the RNA Contained in *Pf*-Derived Vesicles

**Figure 4:**
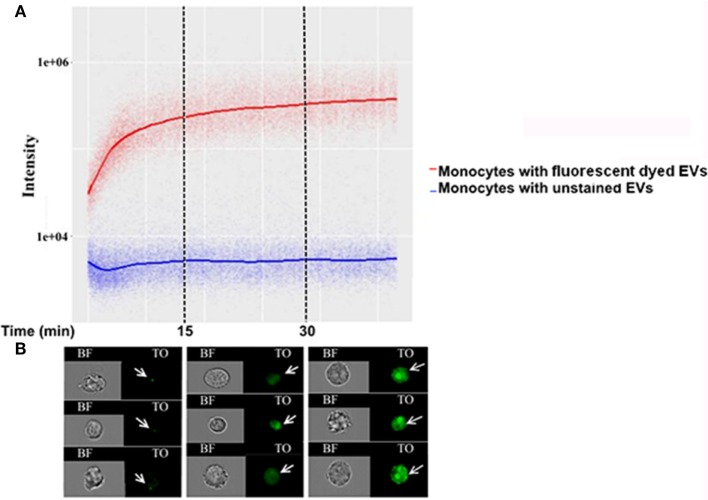
Kinetics measurement of *Plasmodium falciparum* (*Pf*)-extracellular vesicles (EV) uptake into monocytes using imaging flow cytometry (IFC). *Pf*-derived EVs were labeled by thiazole orange (TO), and their uptake into THP-1 cells was measured for 45 min. **(A)** Graph representing the signal modification in TO intensity that was detected over time originating from monocyte recipient cells. Representative results from three independent experiments are shown. **(B)** Signal detected by IFC from three representative recipient cells from each of the three time groups. Within the first 15 min, the EV signal appears as clear spots inside the cells (I), followed by the signal spreading inside the cell area for up to 30 min from EV internalization (II–III).

To understand the *Pf*-EV-cargo’s function in the host target cells, it would be valuable to analyze the kinetics of cargo uptake into target cells. Since we could only detect the lipid signal during the first 5 min of incubation with recipient cells, we examined whether it is possible to explore the uptake kinetics cargo components within the recipient cells over time. This was achieved by establishing a vesicle-uptake kinetics assay. RNA-labeled EVs were added to live THP1 cells and the derived signal was read continuously (after a 90–150 Sec loading time) by IFC for 45 min. A trend line was calculated by the statistical software R, using the “ggplot2” package (36). The smoothing method used was a generalized additive model, which is the package’s default for *n* > 1,000. The results were compared with the acquisition of unlabeled EVs (Figure 4A). As demonstrated, the transferred RNA signal intensity in the cells increased over time, indicating progressive uptake of *Pf*-labeled EVs within monocytes (Figure 4B). Remarkably, the EVs uptake into monocytes occurs rapidly; 10 min after co-incubation most of the monocytes (>90%) stained positive for RNA-cargo (TO dye). Notably, no growth effects were observed within recipient monocytes as compared to control cells during the 72 h post EV uptake (Figure S2 in Supplementary Material).

### Monitoring the IRF3 Translocation to the Nucleus Following *Pf* gDNA Internalization Into Host Monocytes

**Figure 5:**
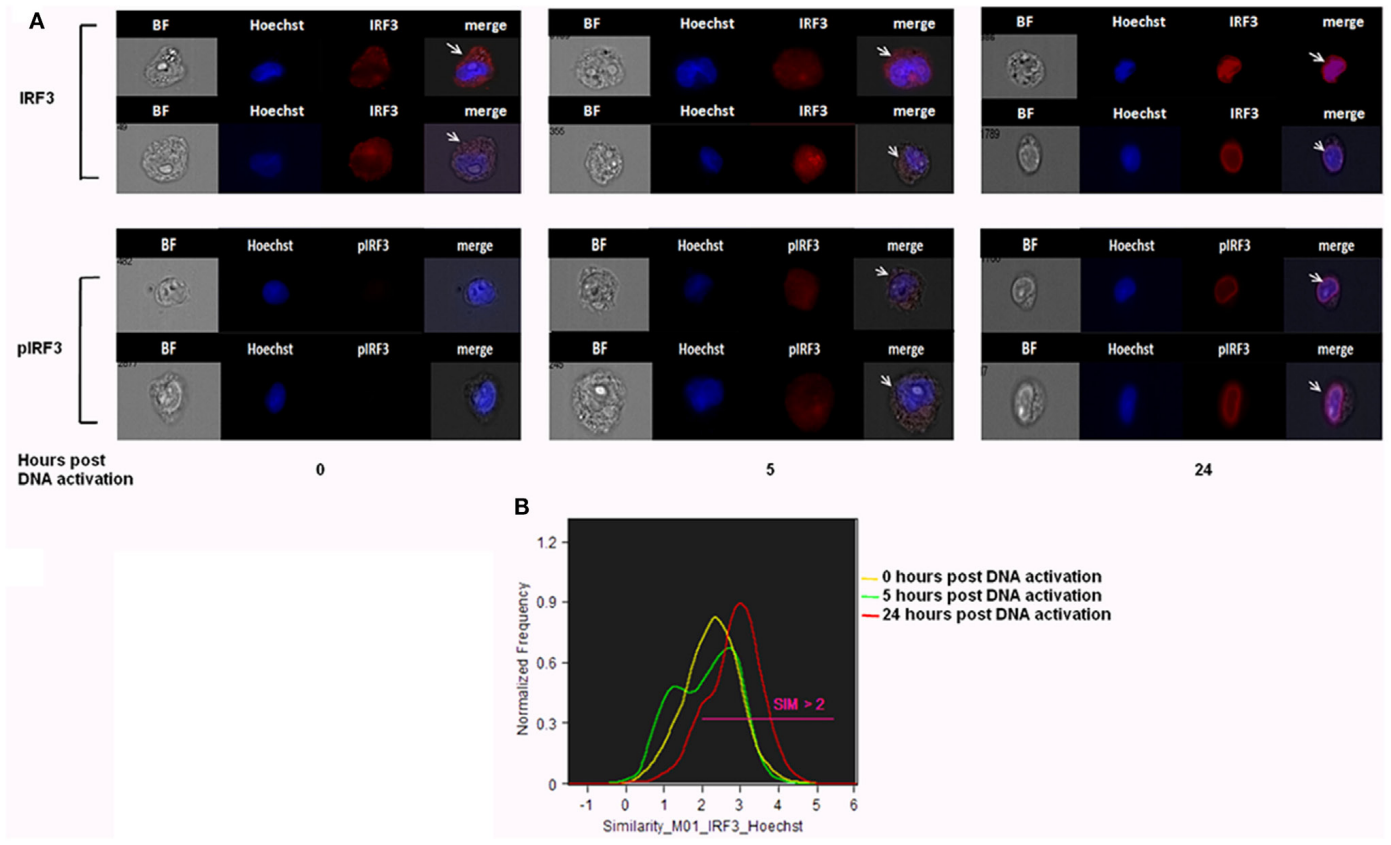
IRF3 and phosphorylated IRF3 (pIRF3) translocation into the nucleus upon *Plasmodium falciparum* (*Pf*)-genomic DNA intake by recipient host monocytes. **(A)** THP-1 cells were transfected with *Pf* gDNA for 5 or 24 h. Cells were next fixated and labeled with Hoechst (DNA dye), IRF3 (upper panel), or pIRF3 (bottom panel) antibodies, imaged and analyzed by imaging flow cytometry at different time points. Representative results from at least three experiments are shown *n* = 2*10^5^. **(B)** Similarity values between the IRF3 and nuclear image. Values over 1.5 are considered as co-localization. Representative results from at least three experiments are shown. *T*-test (*p* < 0.001) analysis for similarity values between 24 h and not treated and between 24 and 5 h was performed.

Previously, we showed that, upon internalization of *Pf* DNA-harboring EVs into host monocytes, the parasitic DNA cargo prompts STING-dependent DNA sensing response. The protein STING subsequently activates kinase TBK1, which phosphorylates the transcription factor IRF3, causing IRF3 to translocate to the nucleus and induce STING-dependent gene expression (16). The ability to track the translocation of proteins within host cells upon pathogen EV uptake could be a useful tool for determining their function and the resultant alteration in signaling pathways within the host cell. We used IFC to test whether it is possible to measure the translocation of transcription factor IRF3 from the cytosol to the nucleus upon insertion of *Pf-*DNA cargo into host cells. For that, monocytes were transfected with *Pf*-genomic DNA that mimics the internalization of parasitic DNA into host monocytes by EVs as described in a previous study (16). Using a specific antibody against the phosphorylated form of IRF3 (pIRF3), we demonstrated that the intensity of the activated form, pIRF3, progressively increased upon the internalization of the cargo (*Pf*-DNA); after 24 h, the majority of pIRF3 was localized in the nucleus (Figure 5A bottom panel). As seen in Figure 5A, upper panel, these results were confirmed by using a primary antibody against IRF3 itself; a positive signal appeared in the nucleus over the course of the 24 h following cargo insertion, indicating the alteration within recipient immune cells and the migration of the transcription factor from the cytosol to the nucleus. Thus, using IFC to track the outcome of *Pf*-EV uptake by host cells may help to reveal the nature of the EV’s role in malaria pathogenesis.

## Discussion

The need for establishing high-throughput EV population characterization methods led us to adapt existing approaches, such as flow cytometry and IFC, for the benefit of the vesicles research field. Measurements using conventional fluorescence microscopy are challenging due to EV fluid dispersal and limited analysis and quantification tools. Conversely, in conventional flow cytometry, objects are measured according to their light scattering and fluorescence intensity, thus limiting the sensitivity for small, dim particles, such as EVs. Reaching a higher dynamic range, lower noise, and a higher quantum yield can be achieved in IFC by using a CCD camera instead of photomultiplier tubes (22–24, 26). In addition, IFC operates in a time delay integration mode, which increases the exposure time from microseconds to milliseconds, further enhancing sensitivity (22–24, 26). By exploiting the 60×, high numerical aperture (NA = 0.9) lens, we reached a high degree of light gathering and sensitivity. When using this lens, the core width is set to 7 µm, making it narrow enough to keep most of the acquired objects in focus. Thus, the combination of precise fluidics and a highly sensitive CCD camera, in addition to careful gating with visual inspection, facilitates the accurate detection of low intensity, small size objects, and making IFC a powerful tool for sensitive, accurate, statistically robust analysis of EVs (24, 27, 28). We also directed our efforts to calibrating the EVs’ double-stain so as to increase the validity of the experimental results. The end results of our efforts was an advanced method for following simultaneously the delivery of different cargo components into recipient cells, which we validated by visually following the internalization of malaria parasitic EVs.

The advantage of the IFC method is that it can be used to study EV uptake in any system, eliminating the need for specific antibodies, but necessitating dependence on non-specific staining (e.g., for RNA, proteins, or lipids). Recent works have indeed successfully used IFC to demonstrate [and a single fluorescent stain (35)] the uptake of vesicles and to characterize the properties of vesicles (24, 28, 29, 34, 35). One study used specific antigens to explore vesicles in blood (37), while another displayed the ability to monitor vesicle adherence in whole blood in a competitive uptake assay (21). Using specific fluorescent-antibodies, the latter study found that vesicles adhere preferentially to monocytes, which supports directed EV targeting. Yet, the mechanisms by which pathogen-derived vesicles are uptaken by target host cells has remained, thus far, mostly elusive.

**Figure 6:**
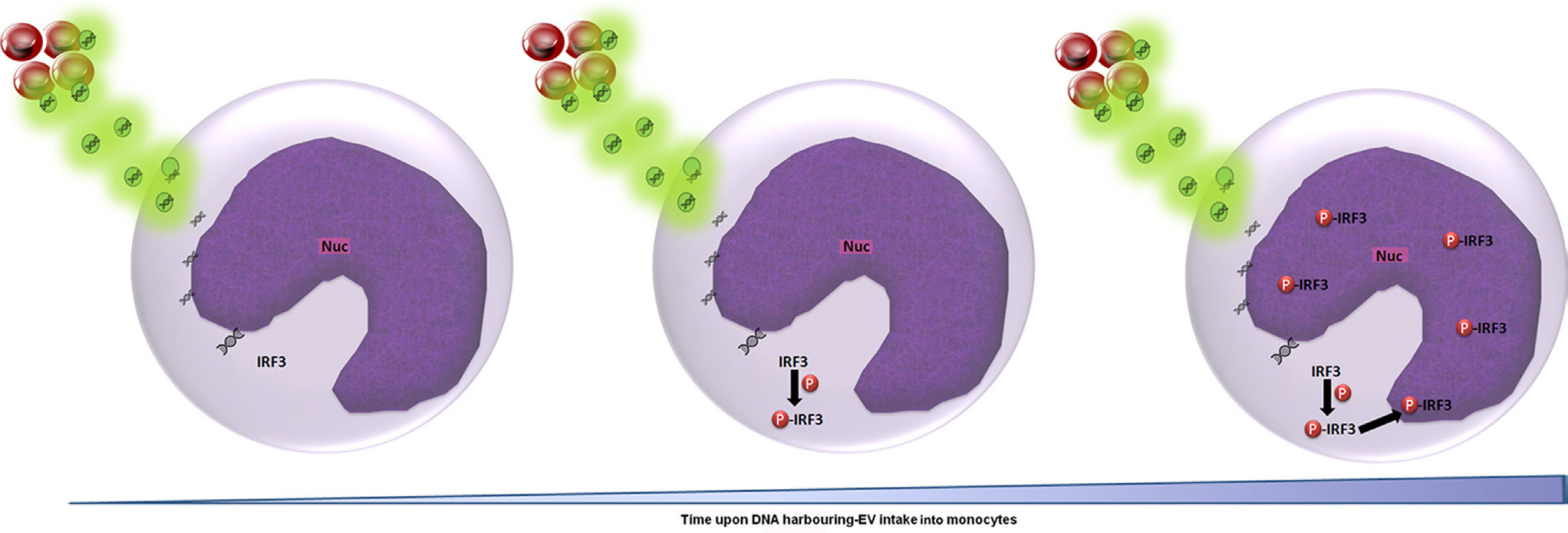
Schematic representation of the cascade of events upon *Plasmodium falciparum* (*Pf*)-gDNA intake by host monocytes. In non-activated monocytes, transcription factor IRF3 is localized in the cytoplasm. Upon *Pf*, DNA cargo internalization into the cytoplasm, IRF3 undergoes phosphorylation and is translocated into the nucleus to induce the cellular immune response (from left to right). Abbreviation: Nuc, nucleus.

Applying the advanced IFC method we developed to the study of malaria parasitic EVs, we successfully isolated EVs derived from *Pf*-infected RBCs and demonstrated their rapid integration (less than 5 min) into human monocytes (Figures 1B, 2B, 3 and 4). Using IFC, we exhibit a robust kinetic assay for measuring cargo internalization during uptake and monitoring the molecular effect of *Pf*-EV cargo internalization into host target cells. We also demonstrated the powerful ability of IFC to directly track the migration of a host transcription factor (IRF3) within the cellular environment once the protein becomes activated due to parasitic cargo internalization (Figures 5 and 6; a schematic illustration). The statistical strength of a robust analysis of thousands of recipient cells increases the physiological feasibility of these occurrences. Characterizing the EV content by different dyes, tracking the kinetics of EV uptake into target cells and, finally, tracking the activation of the specific factors within target cells may shed light on the EVs’ function in host–pathogen communication and, hence, demonstrate the usefulness of IFC as a robust tool to study EV uptake and cargo dynamics.

Such means will open up additional research directions into the cellular alterations of host proteins upon the uptake of pathogen-derived EVs. Therefore, IFC could generally improve our knowledge on EV uptake mechanisms and shed additional light on other EV functions.

## Author Contributions

YO-B, NR-R, and ZP designed the experiments and wrote the paper. YO-B established EV uptake monitoring assay by IFC. YO-B, PK, AR, and TG performed the experiments.

## Conflict of Interest Statement

The authors declare that the research was conducted in the absence of any commercial or financial relationships that could be construed as a potential conflict of interest.

## Supplementary Material

Figure S1 *Plasmodium falciparum* (Pf)-derived extracellular vesicles (EVs) characterization by NTA Nanosight. *Pf*-derived EVs analyzed by Nanosight NS300 (Malvern) for size distribution and particle concentration. The graphs represent the mean of 6*60 s measurements by Nanosight NS300. EV concertation is 3.4*10^7^ ± 1.7*10^7^ and the diameter mean is 91 nm. Representative results from at least three experiments are shown.

Figure S2 THP-1 cell growth following uptake of *Plasmodium falciparum* (Pf)-derived extracellular vesicles (EVs). *Pf*-derived EVs were introduced to THP-1 cells for 5 min, and then washed. **(A)** Cell viability tests. This experiment is a representative of three biological repeats. SD and *T*-test analysis (*p* ≥ 0.1). Representative results from at least three experiments are shown. **(B)** Percentage of dead cells was measured using trypan blue. This experiment is a representative of three biological repeats. SD and *T*-test analysis (*p* ≥ 0.1).

Figure S3 *Pf*-EV intake by monocytes at different temperatures. THP-1 cells were incubated with RNA (TO)-labeled *Pf*-EVs at 37 or 4°C for 5 min. Cells were then washed with ice-cold PBS (–/–) and imaged by imaging flow cytometry. Graphs show TO-labeled positive cells (yellow), gated according to unlabeled cells. At 37°C 37.5% of the cells were positive to TO signal, at 4°C 1.06% of the cells were positive to TO. Abbreviations: TO, thiazole Orange; Pf, *Plasmodium falciparum*; EV, extracellular vesicle.
