# Supplementary figures and images for: Monitoring Extracellular Vesicle Cargo Active Uptake by Imaging Flow Cytometry
Source: Front Immunol. 2018 May 24;9:1011. doi: 10.3389/fimmu.2018.01011 (PMC5976745; doi:10.3389/fimmu.2018.01011)

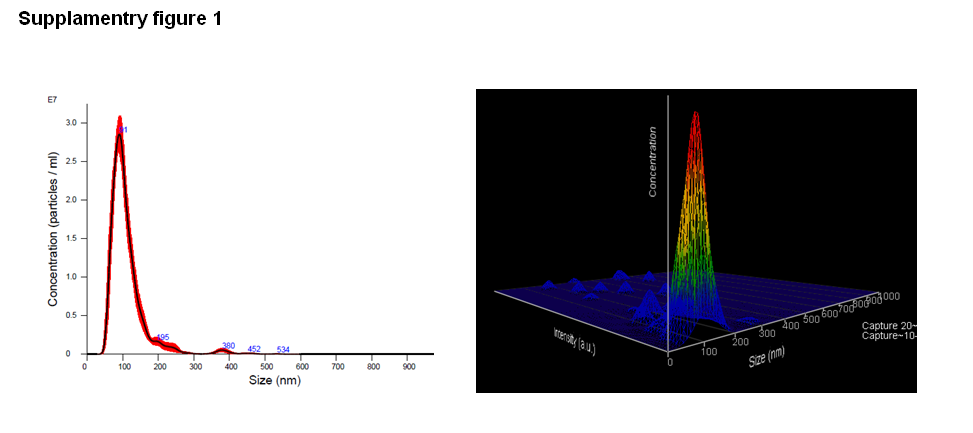

Supplement: Figure S1 — Plasmodium falciparum (Pf)-derived extracellular vesicles (EVs) characterization by NTA Nanosight. Pf-derived EVs analyzed by Nanosight NS300 (Malvern) for size distribution and particle concentration. The graphs represent the mean of 6*60 s measurements by Nanosight NS300. EV concertation is 3.4*107 ± 1.7*107 and the diameter mean is 91 nm. Representative results from at least three experiments are shown. [file image_1.tif]

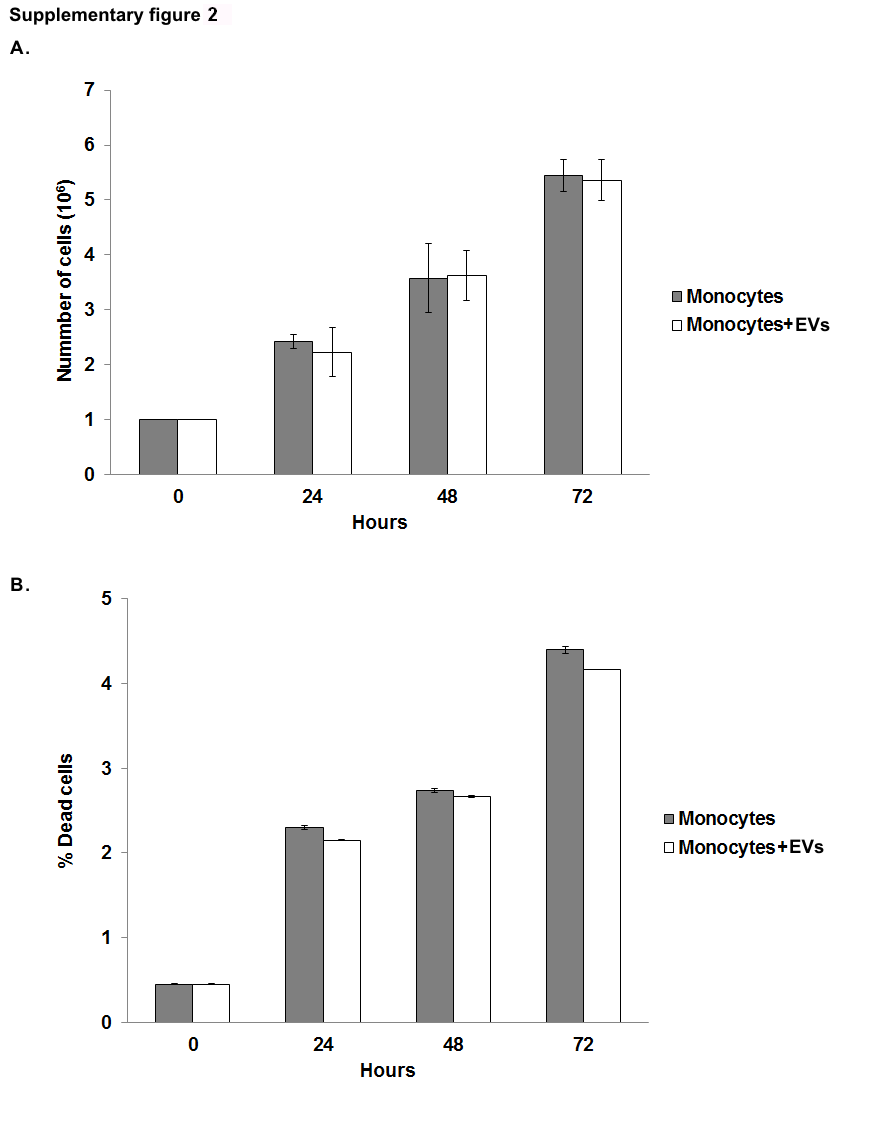

Supplement: Figure S2 — THP-1 cell growth following uptake of Plasmodium falciparum (Pf)-derived extracellular vesicles (EVs). Pf-derived EVs were introduced to THP-1 cells for 5 min, and then washed. (A) Cell viability tests. This experiment is a representative of three biological repeats. SD and T-test analysis (p ≥ 0.1). Representative results from at least three experiments are shown. (B) Percentage of dead cells was measured using trypan blue. This experiment is a representative of three biological repeats. SD and T-test analysis (p ≥ 0.1). [file image_2.tif]

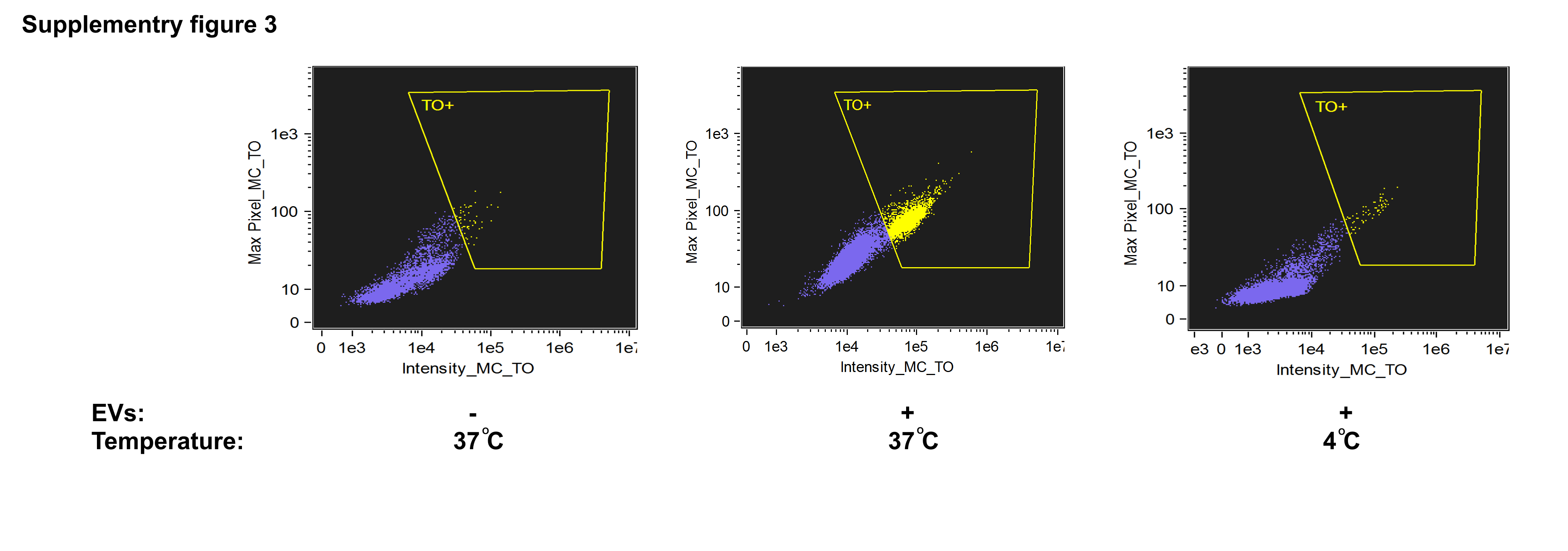

Supplement: Figure S3 — Pf-EV intake by monocytes at different temperatures. THP-1 cells were incubated with RNA (TO)-labeled Pf-EVs at 37 or 4°C for 5 min. Cells were then washed with ice-cold PBS (–/–) and imaged by imaging flow cytometry. Graphs show TO-labeled positive cells (yellow), gated according to unlabeled cells. At 37°C 37.5% of the cells were positive to TO signal, at 4°C 1.06% of the cells were positive to TO. Abbreviations: TO, thiazole Orange; Pf, Plasmodium falciparum; EV, extracellular vesicle. [file image_3.tif]
